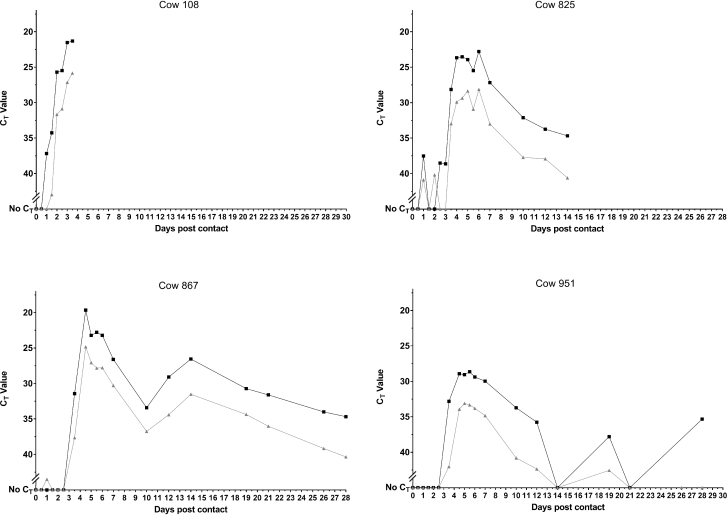# Detection of foot-and-mouth disease virus in milk samples by real-time reverse transcription polymerase chain reaction: Optimisation and evaluation of a high-throughput screening method with potential for disease surveillance

**DOI:** 10.1016/j.vetmic.2018.07.024

**Published:** 2018-09

**Authors:** Bryony Armson, Valerie Mioulet, Claudia Doel, Mikidache Madi, Satya Parida, Karissa A. Lemire, Diane J. Holder, Amaresh Das, Michael T. McIntosh, Donald P. King

**Affiliations:** a The Pirbright Institute, Ash Road, Pirbright, Surrey, GU24 0NF, UK; b Institute of Biodiversity, Animal Health and Comparative Medicine, College of Medical, Veterinary & Life Sciences, Graham Kerr Building, University of Glasgow, G12 8QQ, UK; c Foreign Animal Disease Diagnostic Laboratory, National Veterinary Services Laboratories, Animal and Plant Health Inspection Services, US Department of Agriculture, Plum Island Animal Disease Center, Greenport, NY, 11944, USA

**Keywords:** Foot-and-mouth disease virus, Milk, Real-time RT-PCR, Surveillance

## Abstract

This study aimed to evaluate the utility of milk as a non-invasive sample type for the surveillance of foot-and-mouth disease (FMD), a highly contagious viral disease of cloven-hooved animals. Four milking Jersey cows were infected via direct-contact with two non-milking Jersey cows that had been previously inoculated with FMD virus (FMDV: isolate O/UKG/34/2001). Milk and blood were collected throughout the course of infection to compare two high-throughput real-time reverse transcription polymerase chain reaction (rRT-PCR) protocols with different RT-PCR chemistries. Using both methods, FMDV was detected in milk by rRT-PCR one to two days before the presentation of characteristic foot lesions, similar to detection by virus isolation. Furthermore, rRT-PCR detection from milk was extended, up to 28 days post contact (dpc), compared to detection by virus isolation (up to 14 dpc). Additionally, the detection of FMDV in milk by rRT-PCR was possible for 18 days longer than detection by the same method in serum samples. FMDV was also detected with both rRT-PCR methods in milk samples collected during the UK 2007 outbreak. Dilution studies were undertaken using milk from the field and experimentally-infected animals, where for one sample it was possible to detect FMDV at 10^−7^. Based on the peak C_T_ values detected in this study, these findings indicate that it could be possible to identify one acutely-infected milking cow in a typical-sized dairy herd (100–1000 individuals) using milk from bulk tanks or milk tankers. These results motivate further studies using milk in FMD-endemic countries for FMD surveillance.

## Highlights

• FMDV was detected by rRT-PCR in milk up to 28 days post contact challenge.
• FMDV was detected in milk collected from infected farms in the field (UK 2007).
• FMDV detection was possible when a milk sample was diluted up to 10^−7^ in negative milk.
• Pooled milk has the potential to be a valuable sample type for FMDV surveillance.

## 1 Introduction

Foot-and-mouth disease (FMD) is a highly contagious, transboundary disease of cloven-hooved mammals caused by FMD virus (FMDV) which belongs to the genus *Aphthovirus* within the family *Picornaviridae* (Grubman and Baxt, 2004). Clinical signs of FMD include high temperature, excessive salivation, and the formation of vesicles on the oral mucosa, nose, teats, and the inter-digital spaces and coronary bands of the feet (Kitching, 2002; Alexandersen et al., 2003). FMD is a disease of great economic importance, with an estimated average annual global impact of US $11 billion due to direct losses and the cost of vaccination (Knight-Jones and Rushton, 2013). Consequences of an incursion into a country that is normally free from the disease can be high. For example, the UK 2001 epidemic resulted in the slaughter of over 6 million animals and losses of over £8 billion (Rushton et al., 2002).

Rapid and accurate detection is central to facilitate control and to eventually eradicate the disease. Diagnosis of FMD cases can be carried out using virological, molecular and serological tests (Paton et al., 2009). Real-time reverse transcription polymerase chain reaction (rRT-PCR) assays have been developed with high diagnostic and analytical sensitivity (Shaw et al., 2004). Since they detect viral RNA (or even degraded genome) instead of intact viral antigens and/or live virus, these assays can be used on a number of sample types (Reid et al., 1998, 2003). For FMD diagnosis, sample types submitted to laboratories include epithelial tissue, vesicular fluid, oesophageal-pharyngeal fluid, swabs, and blood or serum. However, some of these invasive collection methods may cause stress to the animal, and commonly require qualified veterinary practitioners to collect the specimens.

Milk is a non-invasive sample type collected from farms on a daily basis and has the advantage that both FMDV and FMDV-specific antibodies can be detected (Burrows et al., 1971; Armstrong, 1997), and has been utilised for surveillance of a number of other diseases (Beaudeau et al., 2001; Heath et al., 2003). Previous experiments have shown that the mammary gland is an organ that is highly susceptible to FMDV replication, and that FMDV can be detected in milk before the appearance of clinical signs (Burrows et al., 1971; Blackwell and McKercher, 1982; Reid et al., 2006). Milk therefore represents a potentially valuable sample source for FMDV detection and surveillance during, and in recovery from a disease outbreak.

Previous studies have investigated FMDV detection by rRT-PCR in milk samples from experimentally infected Holstein-Friesian cattle (Reid et al., 2006) using two-step amplification protocols. This study aimed to build on this previous work, to assess the performance of a more recently developed nucleic acid extraction protocol utilising rapid, higher throughput robotic equipment and newer one-step real-time RT-PCR kits to detect FMDV in milk. Two protocols were compared employing either the TaqMan® Fast Virus 1-Step kit (Applied Biosystems®) (Method A), or the Superscript III Platinum® One-Step qRT-PCR Kit (Invitrogen^™^) (Method B). Jersey cows, producing milk with a high fat content, were used in this study, in order to fully challenge the RNA extraction conditions, which utilised the MagMAX™ Pathogen RNA/DNA Kit (Applied Biosystems®). It is anticipated that the results from this study can be used to support the development of a bulk tank milk surveillance plan (http://securemilksupply.org/) as part of preparedness for combating a possible FMD outbreak in disease-free settings.

## 2 Materials and methods

### 2.1 Experimental samples

*In-vivo* studies were carried out in the high containment unit at The Pirbright Institute, UK and all procedures were licensed by the Home Office (Project Licence number:70/718) and complied with the Animals (Scientific Procedures) Act 1986, EU Directive 2010/63/EU. Four naïve Jersey dairy cows (aged between 2 years, 9 months, and 8 years, 1 month), were infected via direct contact (day 0) with two non-milking Jersey cows that had been inoculated by intra-dermolingual injection with 10^5^ TCID_50_ FMDV O/ME-SA/PanAsia, O/UKG/34/2001 (0.25 mL per inoculation site [n = 2]) two days previously, and that were displaying clinical signs. Animals were observed for clinical signs, and sampled for serum and milk on days -5, to 7, 10, 12, 14, 19, 21, 26 and 28 days post contact (dpc). Milk was collected by machine twice a day until 7 dpc, and once a day thereafter, and daily milk yields recorded by weight. Skimmed milk was separated from the cream and cell fraction by centrifuging an aliquot of each whole milk sample at 3000xg (Hettich Rotanta 460R) for ten minutes.

### 2.2 Field samples

Twelve milk samples collected during the FMDV outbreak in the UK in 2007 (caused by a derivative of FMDV O_1_ BFS 1860) were used to compare diagnostic screening methods (Table 1). These samples were from individual cows displaying clinical signs held at one of the infected premises (IP) 2 (Cottam et al., 2008; Ryan et al., 2008).

**Table 1:** C_T_ values of individual milk samples collected from individual cows obtained from infected premises (IP) 2, from the 2007 UK outbreak of foot-and-mouth disease (FMD) for both methods. (Verification of clinical signs from these animals and formal confirmation of the FMD outbreak was completed by the Pirbright Institute (Ryan et al., 2008)).

Sample ID	Age of oldest lesion	Method A	Method B
c27	Not dated	21.19 (±0.45)	16.50 (±0.28)
105	2 days	21.59 (±0.22)	17.18 (±0.20)
036	5 days	26.18 (±0.17)	22.03 (±0.28)
027	6 days	27.07 (±0.15)	21.46 (±0.15)
369	6 days	24.98 (±0.17)	19.67 (±0.15)
341	6 days	27.15 (±0.14)	21.81 (±0.12)
069	4 days	25.26 (±0.11)	20.15 (±0.20)
030	5 days	27.79 (±0.25)	21.78 (±0.43)
161	2 days	29.58 (±0.08)	24.38 (±0.19)
092	5 days	32.27 (±0.19)	27.94 (±0.30)
241	3 days	22.04 (±0.39)	16.81 (±0.29)
093	5 days	24.64 (±0.27)	20.09 (±0.74)

Data shown are C_T_ values of rRT-PCR performed on three independent extractions for Methods A and B, with standard deviations in parentheses.

### 2.3 Cell culture isolates

FMDV cell culture isolates were obtained from the FMDV repository held at the OIE Reference Laboratory and FAO World Reference Laboratory for foot-and-mouth disease (WRLFMD), Pirbright, UK. Positive controls for rRT-PCR assays were prepared by spiking unpasteurised whole Jersey milk with a 10^−2^ dilution of cell culture isolate O/SAU/1/2016. Analytical sensitivity of the diagnostic screening methods was assessed using a ten-fold dilution series (10^-1^ to 10^-8^) of cell culture isolate A/KEN/6/2012 in whole Jersey milk.

### 2.4 Virus isolation

Virus isolation was carried out on primary bovine thyroid (BTY) cell cultures (Snowdon, 1966), on all experimental samples on the day of collection. Titrations were later performed on milk samples using BTY cell cultures after brief storage at −80 °C, and the viral titre was calculated using the Spearman-Kärber method, as described by the UN, Food and Agriculture Organization (FAO) at http://www.fao.org/docrep/003/v9952e/V9952E02.htm and expressed in units of TCID_50_/mL.

### 2.5 Diagnostic screening methods

Diagnostic screening methods for the detection of FMDV genome in milk samples are defined as Method A and Method B for the purpose of this study, and are described in Table 2. In initial studies, the performance of four RNA extraction and rRT-PCR combinations was assessed. However, some reagents/extraction robots are no longer commercially available or used in diagnostic laboratories, therefore only two methods (A and B) were carried forward for further evaluation in this study.

**Table 2:** Comparison of the two high-throughput foot-and-mouth disease virus detection methods.

	A	B
Extraction kit	MagMAX™ Pathogen RNA/DNA Kit (Applied Biosystems®)	MagMAX™ Pathogen RNA/DNA Kit (Applied Biosystems®)
Internal Control	VetMAX™ Xeno™ Internal Positive Control RNA (Applied Biosystems®)	VetMAX™ Xeno™ Internal Positive Control RNA (Applied Biosystems®)
Sample input	200 μL	200 μL
rRT-PCR kit	‘TaqMan® Fast’ Virus 1-Step Master Mix (Applied Biosystems®)	‘Superscript’ III Platinum® One-Step qRT-PCR Kit (Invitrogen^TM^)
Internal control assay	VetMAX™ Xeno™ Internal Positive Control LIZ™ Assay (Applied Biosystems®)	VetMAX™ Xeno™ Internal Positive Control LIZ™ Assay (Applied Biosystems®)
Primers and Probes	Targeting 3D polymerase (Callahan et al., 2002)	Targeting 3D polymerase (Callahan et al., 2002)
RNA template input	2.5 μL	5 μL

### 2.6 RNA extraction

RNA extractions for both methods were carried out using the MagMAX™ Pathogen RNA/DNA Kit (Applied Biosystems®) on a MagMAX™ Express 96 Extraction Robot (Applied Biosystems®) with a sample input of 200 μL, and elution volume of 90 μL. One μL per reaction of VetMAX™ Xeno™ Internal Positive Control RNA (10,000 copies/μL) (Applied Biosystems®) was also added to the lysis buffer prior to extraction.

### 2.7 rRT-PCR

Two commercially available rRT-PCR kits were evaluated as listed in Table 2. In Method A, the TaqMan® Fast Virus 1-Step Master Mix (Applied Biosystems®) was used with the following thermal cycling conditions: 50 °C for 5 min, 95 °C for 20 s, then 45 cycles of 95 °C for 3 s and 60 °C for 30 s. For this method 2.5 μL of RNA template was added to the rRT-PCR reaction mix containing 6.25 μL of 1-step mastermix (4x, supplied with the kit), 0.25 μL each of forward and reverse primer (20 μM), 0.25 μL probe (10 μM), and 14.5 μL of nuclease free water. In Method B, the Superscript III Platinum® One-Step qRT-PCR Kit (Invitrogen^™^) was performed using the reagents, parameters and thermal cycling conditions previously reported (Shaw et al., 2007), with an RNA template volume of 5 μL. Primers and probes targeting the conserved 3D region of the FMDV genome (Callahan et al., 2002) were used for both methods. This assay has been previously shown to reliably detect viral RNA representing all seven FMDV serotypes (King et al., 2006) and is a widely adopted diagnostic assay recommended by the OIE for use in FMD Reference Laboratories. One μL VetMAX™ Xeno™ Internal Positive Control LIZ™ Assay (Applied Biosystems®) per reaction was also included in the reaction mix. The Applied Biosystems® 7500 Real-time PCR System was used on the ‘fast’ setting for Method A and the ‘standard’ setting for Method B. Evaluation of the RNA extraction and rRT-PCR methods were performed using experimental and field milk samples. Samples were considered positive for all C_T_ values observed until the end of the assay; ≤45 for Method A, and ≤50 for Method B.

### 2.8 Statistics

In order to measure the agreement between the two methods using experimental whole milk samples, Cohen’s Kappa statistic (κ) and the proportion of observed agreement (A*_obs_*) were performed in R version 3.3.3 (R Core Team, 2014) using the package ‘fmsb’, and interpreted as described by Landis and Koch (Landis and Koch, 1977), and linear regression was used to compare C_T_ values. A paired *t*-test was used to compare C_T_ values from both methods using field samples. Unpaired t-tests were used to compare average milk yields before (-6-0 dpc) and during infection (1–6 dpc); both performed in Prism version 7 (GraphPad Software, Inc.).

## 3 Results

### 3.1 Comparison of detection methods with field samples

Twelve milk samples positive for FMDV collected from individual cows during the UK 2007 FMD outbreak were tested using both methods (A and B). Comparisons between the methods demonstrated lower C_T_ values in all samples when using Method B (Table 1) (*p* = <0.001), with a mean C_T_ difference of 5.00 between the two methods. Positive rRT-PCR results were observed in 12/12 (100%) for both methods.

### 3.2 Analytical sensitivity

**Fig. 1:**
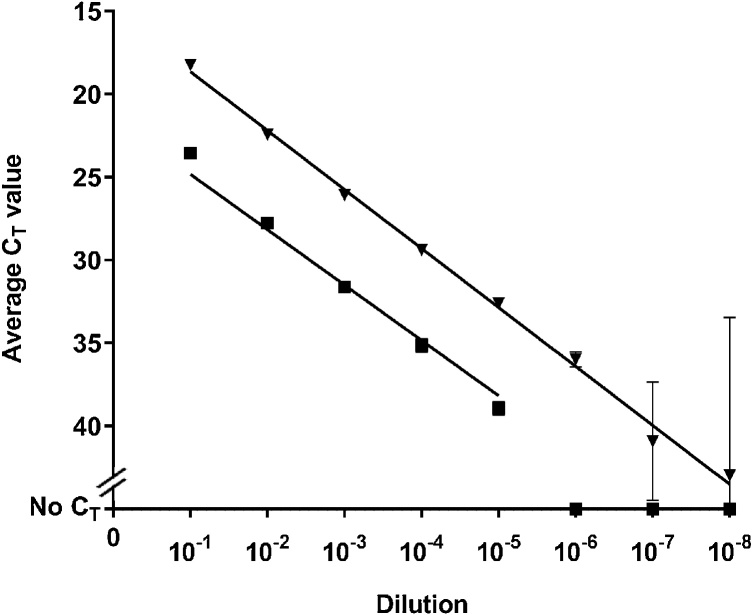
Comparison of the analytical sensitivity for Methods A (used the TaqMan® Fast Virus 1-Step Master Mix (Applied Biosystems®)) and B (used the Superscript III Platinum® One-Step qRT-PCR Kit (Invitrogen^™^)). C_T_ values are the average of three replicates, and bars represent standard deviation. : Method A, : Method B.

The analytical sensitivity of both methods was compared using the ten-fold dilution series of FMD A/KEN/6/2012 spiked into whole Jersey milk (10^−1^ to 10^-8^) (Fig. 1). Without normalizing for different sample input volumes, Method B demonstrated a one log_10_ increase in analytical sensitivity when compared with Method A when all wells were positive, and a range in the difference in average C_T_ value of between 5.33 and 6.30, for Methods A and B. For each dilution, the maximum standard deviation between three technical replicates was 3.55 (Method D, 10^-7^).

### 3.3 Experimental samples

The dairy cows (identified as animal numbers 108, 825, 867 and 951) exhibited clinical signs within 3–4 days after exposure to the inoculated cattle. Cow 108 developed mastitis and was euthanized at 3 dpc, and cow 825 developed a secondary infection and was euthanized at 14 dpc. Both 867 and 951 survived to 28 dpc when the experiment was terminated.

**Fig. 2:**
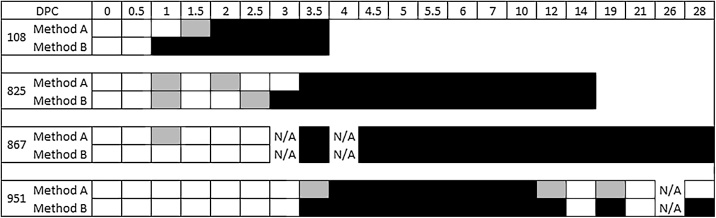
Comparison of both methods tested with experimental whole milk samples. Each square represents the average C_T_ value of the whole milk sample at each day post contact. White squares represent a ‘No C_T_’ value – no detection. Black squares represent any C_T_ value including or below 45 (Method A) or 50 (Method B) in all replicate wells – FMDV positive. Grey squares represent instances where a ‘No C_T_’ value was observed in one or two wells, but a positive result was observed in the other replicates. N/A represents where there was not sufficient sample available for testing.

**Fig. 3:**
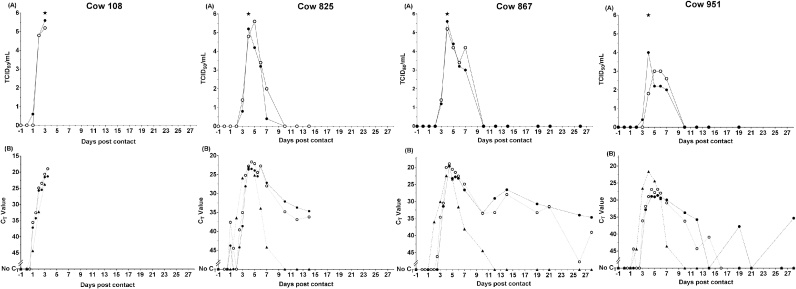
FMDV detection in samples collected at regular intervals from all cows. Virus titrations in BTY cells (A) and rRT-PCR using Method B (B) for skimmed and whole milk fractions and serum (B only). Average C_T_ is derived from the mean of 2 replicates. The development of lesions in at least one foot indicates the onset of clinical signs. : Onset of clinical signs, : whole milk, : skimmed milk, : serum.

Experimental samples were tested with both methods, after a freeze thaw and storage at −80 °C for five years. Based on the testing of 67 whole milk samples, there was agreement (in at least one replicate) between positive and negative results in 61/67 (91.0%) samples across both methods (Fig. 2). When comparing the two methods, almost perfect agreement was observed between the number of positive/negative samples identified (κ = 0.811; *p* = <0.001; A_obs_ = 0.910) (Table 3). Additionally, for the milk samples that were positive using both methods, the average C_T_’s generated were lower when using Method B (R^2^ = 0.704, *p* =  0.001). C_T_ values of the internal controls in all whole milk samples were considered positive by both methods (Method A: mean: 35.37 ± 0.83, Method B: mean: 38.23 ± 2.42). Results from Method B were therefore used to determine the window of virus detection in dairy cows. In most instances at the onset of infection, FMDV detection in milk by rRT-PCR coincided with detection by virus isolation, 1–2 days before the appearance of characteristic foot lesions, and concurrent with the development of nasal discharge in animals 867 and 951. FMDV detection by rRT-PCR in whole milk was observed for animals 108 and 825 until they were euthanised at 3 dpc and 14 dpc respectively (Fig. 3). In addition to early detection, FMDV detection in both milk fractions (whole and skimmed) by rRT-PCR was prolonged, and was extended in whole milk (detected up to dpc 28 for animals 867 and 951), in comparison to virus isolation (detected up to dpc 7 for all three remaining cows). At the onset of infection, rRT-PCR detection of FMDV in serum coincided with FMDV detection in milk, 1 day prior (animals 867, 825 and 951) and the same day (108). In contrast, rRT-PCR FMDV detection in serum ended at 7dpc and 10 dpc, compared to at 28dpc in milk for animals 951 and 867, respectively.

**Table 3:** Comparison of Method A and Method B using experimental whole milk samples.

		Method B		
		Positive^a^	Negative	Total
Method A	Positive^a^	38	2	40
	Negative	4	23	27
	Total	42	25	67
		κ = 0.811; *p* = <0.001; A_obs_ = 0.910		

a Positive results are those with at least one well giving a C_T_ of ≤45 (Method A)/≤50 (Method B).

### 3.4 Impact of FMDV infection on milk yields

Milk yields were recorded by weight on -5 to 6 dpc. The average daily milk yield before cows were infected by direct contact (-6 to 0 dpc) was 22.14 ± 0.51 kg, 20.29 ± 0.45 kg, 18.17 ± 0.86 kg and 18.36 ± 0.43 kg for animals 108, 825, 867 and 951 respectively, these values were used as a baseline to calculate the change in milk yield after infection. The average daily milk yield after infection between days 1–6 dpc, was 23.00 ± 0.58 kg, 22.44 ± 0.82 kg, 16.58 ± 1.96 kg and 15.08 ± 1.59 kg, with an average change of +3.88%, +12.15%, -8.73% and -17.85% for animals 108, 825, 867 and 951 respectively. No significant difference was observed between average yields before and after infection [*p* =  0.356 (108), *p* =  0.450 (867), *p* =  0.056 (951)], apart from for animal 825 [*p* =  0.032 (825)], which demonstrated an increase in average milk yield after infection. The maximum reduction in milk yield recorded on any one day was 50.47% for cow 867, on 6 dpc. The mean difference in milk yield between -6 to 0 dpc and 1 to 6 dpc was greatest for cow 951 (-17.85%, range:-48.26% to +3.49%).

### 3.5 Limit of detection

**Fig. 4:**
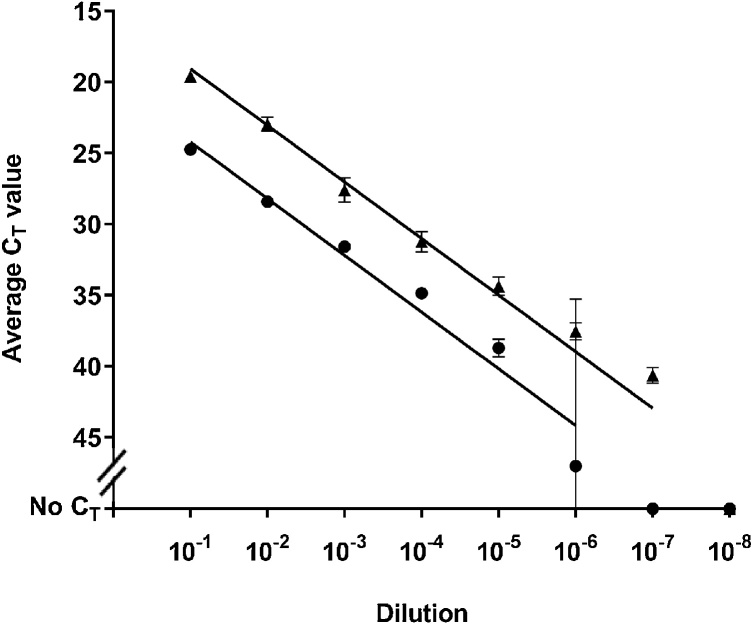
Detection of FMDV by rRT-PCR using Method B on ten-fold dilutions in Jersey whole milk of two milk samples: animal 867 (4.5 days post contact infection) and c27, a field sample from the UK 2007 outbreak (Table 2). C_T_ values are the average of three replicates with standard deviation error bars. : 867 (4.5 dpc), : c27.

To estimate the dilution at which FMDV may still be detected from a pooled milk sample, the limit of detection was determined using the more sensitive Method B, using one milk sample from the animal experiment (867, 4.5 dpc, mean C_T_ value: 19.65) and one milk sample from the 2007 outbreak (animal number c27, mean C_T_ value: 16.50 [Table 1]). Ten-fold serial dilutions were conducted in clean Jersey milk (Fig. 4). Limits of detection were 10^−7^ for sample c27 and 10^-5^ for sample 867 (4.5 dpc) with mean C_T_ values of 40.61 and 38.70 respectively.

## 4 Discussion

Diagnostic sample types of choice for FMD typically comprise vesicular epithelium and vesicular fluid from clinical cases during an outbreak, as they are the richest source of FMDV (OIE, 2013). However, collection of these invasive specimens requires qualified veterinary expertise (Knight-Jones et al., 2016). In contrast, milk is a non-invasive sample type, collected daily, and is utilised for surveillance of a number of other diseases (Beaudeau et al., 2001). This study evaluates two FMDV diagnostic screening protocols utilising high-throughput extraction and rRT-PCR that can be used to gain a diagnostic result in approximately four hours.

Two RNA extraction and rRT-PCR combinations (Methods A and B) were evaluated utilising experimental milk and serum samples, and opportunistic milk samples collected in the field during the UK 2007 outbreak (Ryan et al., 2008). These two methods employ different RT-PCR kits (with different thermocycling conditions) and have been optimised for different RNA template volumes (2.5 μL and 5 μL for Methods A and B, respectively). These specific methods were selected for comparison since they were already used in two of the laboratories that participated in this study. Comparison of these RT-PCR kits using milk samples collected from the UK 2007 outbreak generated lower C_T_ values for all samples with Method B (the MagMax™ Pathogen RNA/DNA kit in combination with the SuperScript™ III Platinum™ One-Step qRT-PCR Kit). It is possible that increasing the RNA template volume for Method A to 5 μL would reduce the number of PCR cycles required to generate signal in the assay; however, the C_T_ differences (i.e., >4) observed in these comparative experiments were greater than would be expected from a two-fold dilution in the volume starting template. Experimental samples were tested by both methods, where more samples were identified as positive using Method B, than Method A, and a greater analytical sensitivity was also observed for Method B using the spiked milk dilution series. Based on these results, Method B was used to determine the window of virus detection during FMDV infection. It was demonstrated that FMDV could be detected in whole milk by rRT-PCR coincident with, and up to 24 days after the onset of early clinical signs of FMD (28 dpc). This was longer than when tested by virus isolation, and for a longer period than with traditional surveillance samples such as serum, from which FMDV was detected only up to six days after the onset of clinical signs. Reid et al. (2006) were only able to detect FMDV RNA up to 23 days post infection, but identified the presence of low copy numbers of FMDV RNA in the mammary lateral lymph node on post-mortem analysis at day 28 post infection. However, for our study, Jersey cattle were used, instead of the Holstein-Friesian cattle that were utilised by Reid et al. (2006), and therefore it is unknown whether this extended detection is due to the higher fat content of the milk from this breed (as FMDV has been shown to be particularly concentrated in the cream component (Reid et al., 2006), or due to the higher analytical sensitivity of the newer detection methods. Ranjan et al. (2016) demonstrated the presence of FMDV in milk samples up to 37 days post clinical manifestation by multiplex (m) PCR and reverse transcription loop-mediated isothermal amplification (RT-LAMP). In this study, animals 867 and 951 were terminated at 28 days post contact, and therefore it is unknown how much longer FMDV RNA might have been detected in these animals. Previous studies have reported FMDV detection up to 51 days post inoculation (Burrows et al., 1971), however this involved the inoculation of FMDV directly into the mammary gland which is not a method of transmission in field situations.

Vesicular lesions on the teats are common in lactating cows with FMD, with infection of the ruptured lesions predisposing animals to the development of secondary mastitis (Kitching, 2002), and field studies have supported this association between FMD and clinical mastitis (Sharma, 2010; Lyons et al., 2015b). During our study, animal 108 displayed lesions on the teats, and animals 108 and 825 developed clinical mastitis (108 and 825), resulting in their euthanasia on days 3 and 14 dpc respectively for welfare reasons. FMDV infection has been shown to cause a reduction in milk yield (Knight-Jones and Rushton, 2013), where secondary mastitis may play a part. However, in our study, when average milk yields were compared before (-6 to 0 dpc) and after (1–6 dpc) infection, no significant decrease was observed, even in cow 108 with secondary mastitis, although the maximum decrease observed on any one day was 50.47% for animal 867. This is comparable to previous experimental studies that demonstrated a maximum reduction of 62.1% on 10 dpc (Reid et al., 2006), and during an outbreak of FMDV in Iran, a total reduction of 8.0% and 4.7% in mean milk production for first and second lactation cows, respectively (Ansari-Lari et al., 2017). These published studies and our study support data reported by Lyons et al. (Lyons et al., 2015a) who observed that although there was a decrease in milk production at the herd level, clinical FMD was shown to be a poor predictor of milk yield, and that no statistical evidence was found to indicate a significant decrease in milk yield between FMD clinical animals and non-clinical cases when lactation curves were modelled.

This study has demonstrated that milk from individual animals could be utilised as a less invasive sample type with simple collection procedures. Pooling these milk samples, or collecting milk from bulk storage tanks would allow for a testing method where there would be no requirement to test all samples individually, thus reducing the cost of testing. Bulk tank milk is used as a sample for a number of other diseases, including bovine viral diarrhoea virus (BVDV) (Renshaw et al., 2000; Hill et al., 2010) and *Coxiella burnetii* (Bauer et al., 2015). In our study, the limit of detection was determined using the better performing Method B, to establish how far a positive milk sample could be diluted in whole Jersey milk and still be detected, simulating the detection of one infected animal from a herd. As expected, the ability to detect FMDV at high dilutions was related to the viral load of FMDV in the individual positive milk, and for one sample, FMDV was detected at a dilution of up to 10^−7^. Based on the peak C_T_ values detected in this study, these findings indicate that it could be possible to identify one acutely-infected milking cow in a typical sized dairy herd (100–1000 individual) using bulk milk sampling. However, further research on the impact of pooling on detection sensitivity is recommended. If virus can be detected in bulk tank milk, this may provide a useful surveillance tool for rapidly detecting infected herds, whilst involving minimal stress to the animal for sample collection. These data may therefore facilitate the design and implementation of surveillance testing plans for FMD in bulk tank milk in readiness for a potential outbreak, or for use in epidemiological studies in endemic regions.

## Conflict of interest statement

The authors have no competing interests to declare.

## Declarations of interest

None.

## Figures and Tables

**Figure:**