# Supplementary material for: Detection of foot-and-mouth disease virus in milk samples by real-time reverse transcription polymerase chain reaction: Optimisation and evaluation of a high-throughput screening method with potential for disease surveillance
Source: Vet Microbiol. 2018 Sep;223:189–94. doi: 10.1016/j.vetmic.2018.07.024 (PMC6127443; doi:10.1016/j.vetmic.2018.07.024)
Supplement: Supplementary file 1 [file mmc1.docx]

Supplementary data 1. Comparison of Methods A and B for the detection of FMDV from whole milk from each cow. C_T_ values are the mean of two replicates.
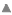
: Method A,
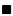
: Method B.
